# Sphingolipid Metabolism in Oral Diseases: Pathogenic Mechanisms, Biomarkers, and Therapeutic Opportunities

**DOI:** 10.3390/biomedicines14081814

**Published:** 2026-08-12

**Authors:** Shixian Zang, Jiaxuan Huang, Ning Duan, Wenmei Wang, Xiang Wang, Qiao Peng, Wei Han

**Affiliations:** Nanjing Stomatological Hospital, Affiliated Hospital of Medical School, Institute of Stomatology, Nanjing University, Nanjing 210008, China; zangshixian715@163.com (S.Z.); h1350016864@gmail.com (J.H.); smallbird@sina.com (N.D.); wenmei-wang@hotmail.com (W.W.)

**Keywords:** sphingolipids, oral squamous-cell carcinoma, periodontitis, oral candidiasis, Sjögren’s syndrome, periapical diseases

## Abstract

Sphingolipids, essential structural components of biological membranes, form a framework that maintains their stability and fluidity. In addition to their structural function, these lipids and their metabolites participate in regulating multiple cellular processes, including proliferation, differentiation, gene expression, and apoptosis, thereby contributing to the maintenance of oral homeostasis. Dysregulation of sphingolipid metabolism is involved in the pathogenesis of several major oral diseases: oral squamous-cell carcinoma (OSCC), periodontitis, oral candidiasis, Sjögren’s syndrome (SS), and periapical diseases. Accordingly, a deeper understanding of sphingolipid biology may provide new opportunities for developing therapeutic strategies targeting these disorders. This review provides a comprehensive analysis of the structural characteristics and principal metabolic pathways of key sphingolipids (e.g., ceramide, sphingosine-1-phosphate [S1P], and glucosylceramide [GlcCer]), discusses their diverse roles in oral diseases, and summarizes recent advances in pharmacological approaches targeting enzymes involved in sphingolipid metabolism.

## 1. Introduction

Sphingolipids, ubiquitous lipids across mammals, fungi, and bacteria, are pivotal membrane constituents that sustain membrane stability, fluidity, and intracellular signal transduction. Technical advances in lipidomics, mass spectrometry, gene knockout models, and specific enzyme inhibitors have accelerated research into sphingolipid metabolism. Core sphingolipids function as both structural components and vital signaling mediators that regulate cell proliferation, differentiation, apoptosis, and inflammation. Disrupted sphingolipid homeostasis drives the onset and progression of numerous diseases.

The oral mucosa, a critical physical barrier, is colonized by abundant microorganisms, with oral homeostasis maintained via mucosal immunity and microbial equilibrium. Emerging evidence implicates aberrant sphingolipid metabolism in the pathogenesis of diverse oral disorders: dysregulated sphingolipid signaling mediates OSCC progression/metastasis, periodontal inflammatory bone loss, oral candidiasis virulence, salivary gland injury SS, and periapical tissue destruction.

This narrative review outlines sphingolipid structures, metabolic cascades, and biological functions, focuses on their regulatory roles in oral diseases, and further discusses sphingolipid-based diagnostic biomarkers and sphingolipid-targeted therapeutics. Five representative oral pathologic conditions—neoplastic, inflammatory bone, fungal, autoimmune, and endodontic periapical diseases, all with well-documented links to sphingolipid metabolism—are analyzed to establish a framework for elucidating oral disease mechanisms and identify promising directions for novel diagnostic and treatment approaches. The relevant literature was retrieved from PubMed and Web of Science using disease-specific keywords, with an emphasis on studies published over the past 15 years; reference lists of included articles were additionally screened.

## 2. Sphingolipids

Sphingolipids, essential structural components of mammalian cell membranes and bioactive signaling molecules, regulate cell proliferation, differentiation, apoptosis, and inflammation. All sphingolipids contain a common sphingoid base backbone conjugated to a fatty acid through an amide bond to form ceramide, which serves as the central structural and metabolic hub of this lipid family [1]. Attachment of various polar head groups to ceramide yields structurally diverse derivatives, including sphingomyelin (SM), GlcCer, S1P, and ceramide-1-phosphate (C1P) [2]. Among these sphingolipids, ceramide and S1P function as opposing pivotal bioactive mediators of cell fate: ceramide predominantly promotes apoptosis and growth arrest, whereas S1P signals through five G-protein-coupled receptors (S1PR1–5) to promote cell survival, proliferation, and migration [1,3].

**Figure 1:**
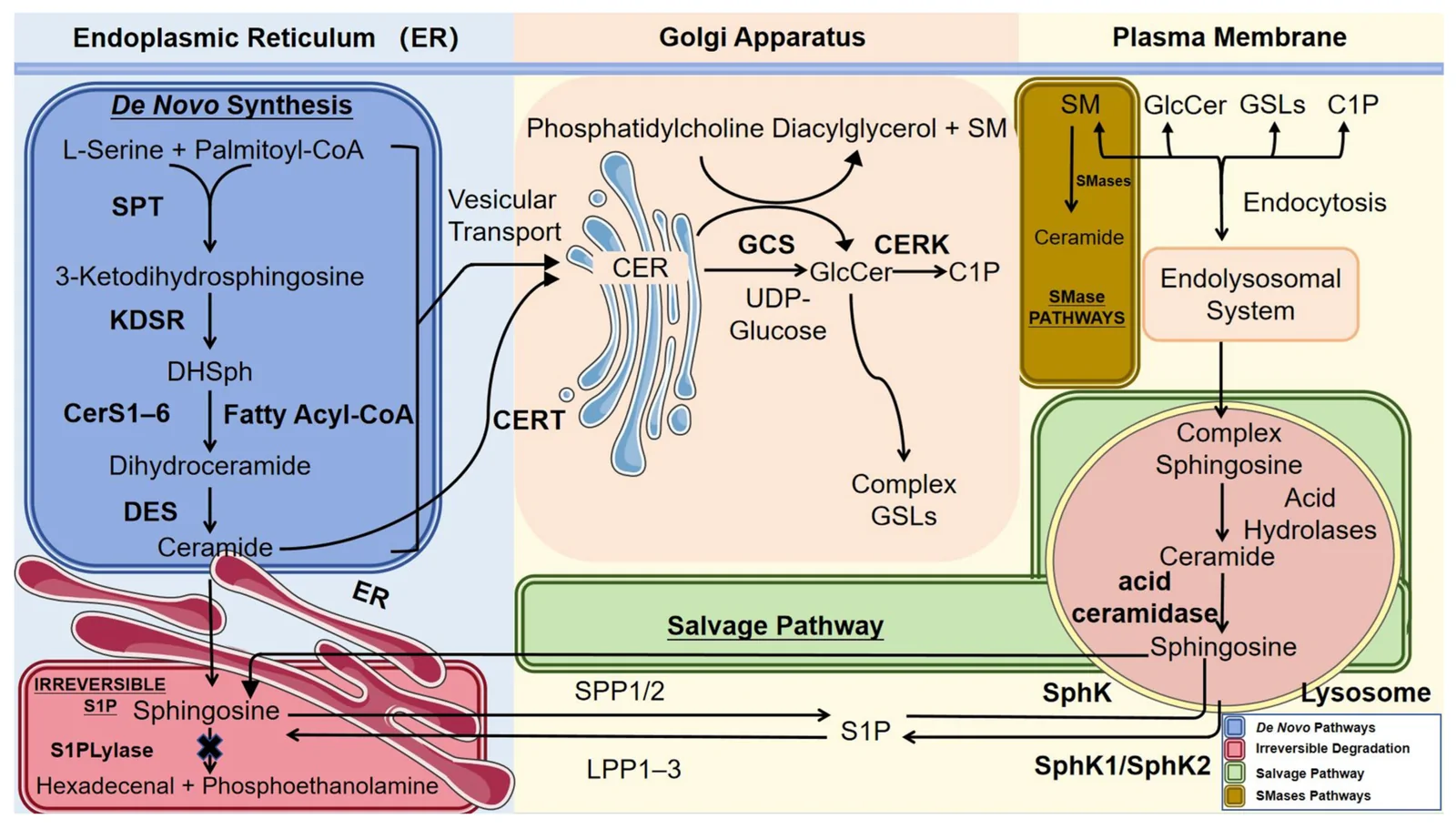
Sphingolipid Metabolic Pathways. Schematic overview of mammalian sphingolipid metabolic network. Four core metabolic branches are color-coded and clearly demarcated: de novo synthesis (blue, endoplasmic reticulum), salvage pathway (green), sphingomyelinase (SMase) degradation pathways (orange), and irreversible S1P catabolism (red). Key metabolic enzymes, intermediate lipids and subcellular compartments (ER, Golgi apparatus, plasma membrane, endolysosome/lysosome) are labeled. DES corresponds to dihydroceramide desaturase (DEGS1) as defined in the main text. Abbreviations: SPT, serine palmitoyltransferase; CerS, ceramide synthase; CERT, ceramide transfer protein; GCS, glucosylceramide synthase; CERK, ceramide kinase; SphK, sphingosine kinase; LPP, lipid phosphate phosphatase; S1PLyase, S1P lyase; SMase, sphingomyelin.

Sphingolipid metabolism is compartmentalized between the endoplasmic reticulum (ER), Golgi apparatus, and endolysosomal system (Figure 1). De novo sphingolipid synthesis initiates at the ER with condensation of serine and palmitoyl-CoA by serine palmitoyltransferase (SPT), followed by reduction and N-acylation catalyzed by six ceramide synthases (CerS1 to CerS6) to generate dihydroceramide, which is desaturated by dihydroceramide desaturase (DES) to ceramide [4,5,6,7,8]. Ceramide is transported to the Golgi apparatus via vesicles or the transfer protein (CERT), where it is converted to SM, GlcCer, or C1P [1,4,9]. Catabolism proceeds in the endolysosomal system, where membrane sphingolipids are internalized and sequentially degraded to ceramide, which is then hydrolyzed by acid ceramidase to sphingosine and subsequently phosphorylated by sphingosine kinase (SphK) to generate S1P. S1P is irreversibly degraded by S1P lyase, representing the sole terminal exit point of the sphingolipid metabolic network [3,10,11,12,13].

The tissue-specific expression of CerS1–CerS6 determines the acyl-chain composition of cellular ceramide pools, influencing cell fate [14,15]. SphK, the enzyme responsible for S1P production, exists as two isoforms—SphK1 and SphK2—with distinct subcellular distributions and biological functions. SphK1 is predominantly cytosolic and translocates to the plasma membrane upon activation to promote extracellular S1P release; its overexpression confers chemoresistance in multiple cancer types [16,17,18,19]. SphK2 exhibits context- and localization-dependent functions: low-level overexpression drives cell proliferation through plasma membrane enrichment and increased extracellular S1P secretion, whereas ER- or mitochondria-localized SphK2 increases ceramide levels to trigger apoptotic signaling [20,21]. Other clinically relevant enzymes include glucosylceramide synthase (GCS), which converts ceramide to GlcCer, and acid ceramidase, both of which reduce cellular ceramide levels and promote chemoresistance in cancer [22,23].

Disruption of sphingolipid metabolic homeostasis in oral tissues may lead to abnormal activation of these pathways, triggering the development and progression of various oral diseases. Among these, the molecular mechanisms involving sphingolipid signaling have been most extensively investigated in OSCC and periodontitis. Therefore, this review summarizes current knowledge regarding the role of sphingolipids in oral diseases and provides an integrated overview of their potential diagnostic and therapeutic relevance.

## 3. Contributions of Sphingolipids and Their Metabolites to Oral Disease Pathophysiology

### 3.1. Sphingolipids and OSCC

OSCC is the most common malignant tumor in the oral cavity, constituting roughly 90% of all oral malignancies worldwide [24,25]. Epidemiological data showed that oral cancer contributed 377,713 newly diagnosed cases globally in 2020, and this incidence figure is projected to exceed 415,000 by 2025. To date, the global five-year survival rate of oral cancer patients remains merely 50–60% [26,27]. The occurrence of OSCC is driven by a variety of pathogenic factors. Among these triggers, tobacco smoking and alcohol intake serve as the dominant risk determinants, especially prevalent in Western populations.

#### 3.1.1. Molecular Mechanisms of Dysregulated Sphingolipid Metabolism in Regulating OSCC Onset and Progression

The onset and progression of OSCC are closely associated with dysregulation of sphingolipid metabolism, which involves multiple molecular mechanisms, including alterations in key metabolic enzymes, intracellular signaling pathways, and lipid metabolic homeostasis.

SphK1, a key enzyme in sphingolipid metabolism, is highly expressed in OSCC [28]. The expression level of SPHK1 is closely correlated with tumor stage, lymph node metastasis, and poor prognosis. Cellular and nude xenograft tumor studies have demonstrated that SPHK1 promotes the proliferation, migration, and invasion of OSCC cells while inhibiting apoptosis by activating the NF-κB/p65 signaling axis and modulating EMT-related molecules, establishing SPHK1 as a critical oncogenic target governing the malignant progression of OSCC [29]. In addition to S1PR1, S1PR2 is also involved in OSCC progression. S1PR2 functions as an important mediator of epidermal growth factor (EGF)-induced migration and invasion of OSCC cells, as inhibition of S1PR2 markedly attenuates the pro-metastatic effect of EGF [30]. EGF can simultaneously increase S1P production by upregulating SphK1, thus forming a feedback mechanism that further activates S1PR2-dependent signaling [30].

Regarding S1P degradation, Vishwakarma et al. recently showed that lipid phosphate phosphatase 3 (LPP3), which dephosphorylates S1P to sphingosine, is significantly downregulated in OSCC tissues compared to non-dysplastic oral mucosa, and low LPP3 levels independently predict perinodal extension, lymphovascular invasion, and higher TNM stage [31]. Nevertheless, SphK2 exerts context-specific functions in oral carcinogenesis. Fugio et al. further validated that high accumulation of SphK2 alone can trigger malignant transformation in normal oral keratinocytes, while forced SphK2 overexpression amplifies the tumorigenic capacity of established OSCC cell lines [32]. These apparently contradictory findings likely reflect the established ability of SphK2 to exert opposing functions, depending on its subcellular localization and cell type [33]. However, the precise localization-dependent roles of SphK2 remain uncharacterized in OSCC tissues. In contrast to SphK1, which is widely studied in different types of cancer, including head and neck cancer, the role of SphK2 in the development and progression of OSCC is still poorly understood. Notably, SphK2 accumulation represents an early event in oral carcinogenesis [32], highlighting SphK2 as a promising therapeutic target for OSCC treatment. The biological roles of other S1P-catabolizing enzymes and S1P transporters in oral cancer have yet to be fully elucidated.

In addition to signaling crosstalk between key enzymes and receptors, the dysregulation of the ceramide/S1P axis represents another important mechanism contributing to OSCC progression.

Dysregulation of the ceramide/S1P rheostat in OSCC is governed not merely by total ceramide abundance, but also by acyl-chain length, the specific ceramide isoform, and subcellular localization. Ceramides constitute a structurally diverse lipid family whose biological activities are dictated by the fatty acyl chain bound to the sphingoid scaffold. In HNSCC, CerS1-synthesized C18-ceramide consistently functions as a tumor suppressor, with reduced C18-ceramide levels correlating with lymphovascular invasion, lymph node metastasis, and advanced tumor stage [34]. In contrast, CerS6-derived C16-ceramide exhibits highly context-dependent bioactivity. At the cellular level, CerS6 expression is drastically suppressed in cisplatin-resistant OSCC cells; restoration of CerS6 expression reinstates mitochondrial fission, suppresses cytoprotective autophagy, and restores cisplatin sensitivity in resistant tumor cells [35]. Separately, systemic C16-ceramide depletion induced by neuroendocrine stressors such as depression also accelerates OSCC progression in vivo through attenuated mitochondrial apoptosis, a phenotype fully rescued by exogenous C16-ceramide supplementation [36]. Collectively, these findings demonstrate that both C18- and C16-ceramide exert tumor-suppressive effects in OSCC under distinct cellular and systemic conditions. Instead of the buildup of a single oncogenic ceramide variant, loss of these two anti-tumor ceramide pools shifts the ceramide/S1P rheostat toward a pro-tumor phenotype.

In addition to the balance between individual ceramide species, tumor cells use multiple enzymatic strategies to reduce the total intracellular ceramide pool. First, Cer is hydrolyzed by ceramidases to sphingosine, which is subsequently phosphorylated by SphK1 to generate the pro-survival mediator S1P [22]. Second, ceramide is glycosylated to GlcCer by GCS. In OSCC, GCS is significantly overexpressed in tumor tissues, and elevated GCS expression independently predicts poor locoregional control and decreased disease-free survival [37], confirming that augmented conversion of ceramide to GlcCer represents a clinically relevant therapy resistance mechanism. Third, ceramide is phosphorylated to C1P by ceramide kinase, which can drive pro-inflammatory signaling [38]. In addition to these canonical evasion routes, Zhao et al. recently showed that dihydroceramide desaturase (DEGS1)—the enzyme that converts dihydroceramide to ceramide—is significantly upregulated in OSCC by spatial metabolomics, with high DEGS1 expression independently predicting poor survival [39], suggesting that altered ceramide desaturation may also contribute to OSCC pathogenesis. Collectively, OSCC cells evade apoptotic signaling and develop cross-resistance to chemotherapy and radiotherapy through two coordinated metabolic shifts: impaired ceramide synthesis driven by diminished CerS1 and CerS6 expression, and accelerated ceramide catabolism mediated by elevated GCS and SphK1 activity. Combined with perturbed lipid desaturation governed by DEGS, these quantitative and qualitative changes collectively deplete cellular pro-apoptotic ceramide reserves.

Although several aspects of sphingolipid metabolism in OSCC have been elucidated, many critical mechanisms remain to be clarified. For example, although DEGS1 has been identified as a pivotal enzyme linking ceramide metabolism to OSCC progression [39], the full spectrum of ceramide synthase and desaturase isoforms, their substrate specificities and downstream lipid mediators in OSCC remains systematically uncharacterized. The role of systemic ceramide fluctuations, driven by neuroendocrine factors such as depression [36], in OSCC risk and progression also merits further epidemiological and mechanistic investigation. Moreover, the biological significance of bacterial sphingolipids within the oral tumor microenvironment and their potential crosstalk with host ceramide metabolism represent an emerging largely unexplored area [40].

#### 3.1.2. Screening and Application of Sphingolipid Markers for OSCC Diagnosis and Prognosis

OSCC often presents with insidious early symptoms and lacks reliable screening indicators, highlighting the need for specific biomarkers for early diagnosis and prognostic evaluation. In this context, molecules involved in sphingolipid metabolism have attracted increasing research attention as potential biomarkers.

SphK1 is the most extensively studied isoform. Hou et al. demonstrated that SPHK1 is significantly overexpressed in OSCC tissues, with expression levels correlating positively with clinical stage, tumor size, pathological grade, and lymph node metastasis; high SPHK1 independently predicted reduced overall survival in a cohort of 54 OSCC patients [29]. In vivo, SphK1 knockout mice exhibited markedly reduced 4-NQO-induced tongue carcinogenesis [28], highlighting SphK1′s role in oral tumorigenesis.

Ceramide species and their metabolic enzymes have also shown discriminative potential for OSCC assessment. Reduced C18-ceramide is associated with lymphovascular invasion, nodal metastasis, and advanced stages in HNSCC [34]. Moreover, salivary CERS1 levels decline progressively from healthy controls through oral leukoplakia to OSCC [41], indicating non-invasive screening potential. GCS, which converts pro-apoptotic ceramide to GlcCer, is overexpressed in OSCC and independently predicts poor locoregional control and disease-free survival [37]. Regarding S1P metabolism, LPP3 is downregulated in OSCC, and reduced LPP3 independently predicts perinodal extension, lymphovascular invasion, and higher TNM stage [31]. Conversely, S1PR3 overexpression correlates with adverse clinicopathological features and poor prognosis [42].

The most comprehensive lipid profiling to date, by Faedo et al., identified a plasma panel (d18:0 S1P + C16:0 ceramide-1-phosphate) with 100% discriminatory accuracy, indicating that C24:0 ceramide and C24:1 GlcCer are independent prognostic factors in OSCC [43]. At the spatial level, Zhao et al. identified DEGS1 as a spatially resolved marker whose high expression independently predicts poor survival [39].

Most studies to date have reported differential expression rather than validated diagnostic performance. Except for Faedo et al. [43], who provided sensitivity and specificity data, most candidate markers await formal validation with ROC analysis in independent, prospectively collected cohorts.

#### 3.1.3. Sphingolipid-Targeted Therapeutic Strategies: Preclinical Evidence

From a therapeutic perspective, elucidating oncogenic mechanisms in OSCC may facilitate the development of targeted strategies that reduce drug resistance and minimize toxic side effects. Molecular targeted therapy provides alternative options for patients unable to tolerate conventional chemotherapy or radiotherapy, particularly through interventions targeting sphingolipid metabolic pathways.

SphK1 mediates chemoresistance in OSCC cells; pharmacological inhibition or gene silencing of SphK1 suppresses OSCC progression and restores radiosensitivity in radioresistant OSCC cell lines (e.g., SCC-15 and SCC-25) [44,45]. SphK2 has likewise emerged as a therapeutic target. Liu et al. developed QZQ-01115, a novel SphK2 inhibitor that suppressed CAL-27 cell proliferation (IC50 = 5.84 μM) by disrupting the S1P/ceramide balance and downregulating PI3K/AKT/mTOR signaling, ultimately inducing G0/G1 cell cycle arrest and apoptosis via Bcl-2 downregulation and Bax upregulation; its in vivo anti-tumor efficacy was subsequently confirmed in a xenograft model [46]. Roh et al. further demonstrated that acid ceramidase overexpression confers cisplatin resistance in HNSCC, and its inhibition via N-oleoylethanolamine or gene silencing may reverse chemoresistance, suggesting that concomitant administration of acid ceramidase inhibitors with cisplatin may overcome drug resistance [23].

Regarding S1P receptor targeting, S1PR2 inhibitors (e.g., JTE013) may enhance EGFR-targeted therapy and suppress OSCC metastasis [30]. S1PR3, another S1P receptor subtype, is overexpressed in OSCC and associated with poor prognosis; pharmacological inhibition of CAY10444 or genetic knockdown impairs the AKT/WEE1 cell cycle checkpoint and suppresses OSCC proliferation both in vitro and in vivo [42]. FTY720, an S1PR modulator approved for multiple sclerosis, has recently been evaluated in OSCC. Torres et al. demonstrated that FTY720 combined with paclitaxel reduced viability, clonogenicity, and cancer stem cell sphere formation in cisplatin-resistant OSCC cells (Cal27 and SCC9), and significantly inhibited xenograft tumor growth by suppressing NFκB/SET/SphK1 signaling [47].

Short-chain ceramide analogs also hold therapeutic promise. Zhu et al. reported that C6-ceramide (10 μM) combined with PKC412 synergistically enhanced anti-tumor effects by inhibiting the Akt-mTOR pathway and downregulating Mcl-1 [48]. Liposomal C6-ceramide formulations have been engineered to address ceramide delivery constraints, providing experimental evidence for potential clinical translation [49].

In addition, to further optimize the bioavailability and tumor selectivity of such lipid agents, nanoparticle-based delivery systems, such as gold nanorod-mediated SphK1-siRNA delivery and ceramide nanoliposomes, have been explored to improve drug delivery efficiency and tumor targeting specificity [50].

In summary, key enzymes (SphK1 and SphK2), lipid mediators (ceramide and S1P), and receptors (S1PR2 and S1PR3) in sphingolipid metabolism contribute to diagnostic and prognostic biomarker development for OSCC and provide multiple therapeutic targets for precision medicine approaches aimed at tumor progression, drug resistance, and metastasis (Table 1). These advances may support future translational research and clinical development of targeted therapies for OSCC.

### 3.2. Sphingolipids and Periodontitis

Periodontitis, a chronic inflammatory disease primarily triggered by bacterial pathogens affecting the tooth-supporting tissues, is characterized by progressive inflammation and destruction of periodontal structures, ultimately leading to tooth loss [51,52]. Previous studies have indicated that periodontal diseases are associated with systemic conditions such as diabetes mellitus, preterm birth, and atherosclerotic cardiovascular diseases [53,54,55]. Increasing evidence suggests that sphingolipids may play an important role in the pathogenesis of periodontitis.

#### 3.2.1. Sphingolipid Markers for Periodontitis Diagnosis and Prognosis

Early diagnosis of periodontal disease remains challenging, and the development of cost-effective diagnostic strategies and therapeutic approaches continues to be an important research focus [56]. There is an urgent clinical need for reliable biomarkers that enable early detection of periodontal disease, although no widely accepted laboratory diagnostic markers have yet been established [57]. Ceramide phosphoethanolamine (CPE) and its dihydrogenated form dihydroceramide phosphoethanolamine (DH-CPE) are present in *Bacteroidetes* species and in the dental plaque of patients with periodontal disease [58,59,60]. Fluorescently labeled erylysin A (EryA-mCherry) specifically binds to CPE without cross-reacting with host SM, thereby enabling discrimination between healthy individuals and patients with periodontitis, thus providing a basis for early non-invasive diagnosis [61]. In addition, several specific SM species (including d17:1/14:0, d16:1/15:0, and d18:1/21:0) show positive correlations with periodontitis and negative correlations with gingivitis, suggesting their potential value in the differential diagnosis of periodontal diseases [62]. Furthermore, phosphorylated dihydroceramide is detected at high levels in diseased teeth and subgingival plaque samples from patients with periodontitis, supporting its potential use as a diagnostic biomarker [63]. Overall, the distinctive biochemical characteristics of sphingolipid molecules may provide promising opportunities for the development of diagnostic biomarkers for periodontal disease (Table 2).

#### 3.2.2. Molecular Mechanisms of Dysregulated Sphingolipid Metabolism in Regulating Periodontitis Onset and Progression

##### Host-Derived Sphingolipids

Ceramide, a core bioactive molecule in sphingolipid metabolism, enhances the release of inflammatory mediators in periodontal tissues by activating inflammatory signaling pathways such as NF-κB and p38/JNK [64]. In addition, it inhibits mitochondrial oxidative phosphorylation, thereby increasing ROS production and exacerbating lipotoxicity-mediated cellular damage [65]. Ceramide species induce apoptosis in periodontal ligament stem cells and gingival fibroblasts [66]. Under lipotoxic conditions, C16-ceramide accumulates in bone through de novo synthesis, promoting osteoclast formation and suppressing osteoblast mineralization simultaneously; pharmacological blockade of ceramide synthesis by myriocin rescues this mineralization suppression [67,68]. In contrast, extracellular ceramide binding to the inhibitory immunoreceptor CD300lf suppresses STAT3/AKT/ERK phosphorylation, downregulates NFATc1, c-Fos, CTSK, and Acp5, reduces TRAP-positive multinucleated osteoclasts, and attenuates alveolar bone resorption in a ligature-induced mouse model (the interaction of CD300lf and ceramide reduces the development of periodontitis by inhibiting osteoclast differentiation). These divergent outcomes reflect differences in ceramide species (C16-ceramide vs. long-chain ceramide), subcellular localization (intracellular vs. extracellular), receptor signaling pathway (cathepsin B/RANKL vs. CD300lf), and experimental model systems (in vitro vs. ligature-induced periodontitis in vivo) [66]. The deficiency of acid SMase (ASMase) leads to abnormal ceramide accumulation by stimulating the de novo ceramide synthesis pathway, thus exacerbating LPS-induced experimental periodontitis. These findings suggest that ASMase plays an important regulatory role in maintaining periodontal inflammatory homeostasis and bone metabolic balance [69].

Jin et al. reported that elevated serum palmitic acid levels in patients with diabetes or metabolic syndrome, in combination with LPS released by periodontal pathogens, synergistically activate sphingolipid metabolic pathways and promote S1P production through the SphK1-mediated synthesis [70]. Consistent with these mechanistic findings, Hamdan et al. demonstrated that SphK1 protein levels and S1P concentrations significantly increase in gingival crevicular fluid sampled from periodontitis-affected sites compared to healthy controls, with both declining following non-surgical periodontal treatment, providing direct clinical evidence for SphK1/S1P pathway activation in inflamed human periodontal tissues [71]. S1P subsequently enhances the secretion of pro-inflammatory cytokines, including IL-6, by binding to S1PR1 and S1PR2 on macrophages, thereby establishing an inflammatory amplification loop that exacerbates periodontal tissue destruction [70]. Low concentrations of S1P also promote the chemotactic migration of osteoclast precursors toward inflammatory periodontal sites. In contrast, higher concentrations activate S1PR2 and induce a repulsive effect, thus influencing bone resorption by regulating osteoclast precursor recruitment [72]. The functional relevance of this mechanism was supported by Lee et al., who reported that the S1PR1-binding molecule FTY720 reduced osteoclast formation in a rat ligature-induced periodontitis model by promoting osteoclast precursor re-migration from the alveolar bone surface into the vasculature, providing preclinical evidence that S1PR1 targeting may mitigate inflammatory bone resorption in periodontitis [73]. Additionally, S1P modulates Th17/Treg balance and macrophage polarization (M1/M2), promoting inflammatory bone resorption and contributing to the progression of periodontitis [74,75,76]. At the systemic level, a large population-based study showed that serum S1P concentrations were significantly associated with periodontitis severity and extent, with increased expression of SphK1 in inflamed gingival epithelium and CD68^+^ macrophages, further supporting S1P as a systemic inflammatory mediator linking periodontitis to chronic inflammation [77].

**Table 2:** Sphingolipid Biomarkers for Periodontitis Diagnosis and Prognosis.

Biomarker/Molecule	Sample Type	Key Finding	Clinical Potential	Refs.
CPE/DH-CPE	Dental plaque	Present in *Bacteroidetes* spp. and periodontitis patients; absent in healthy controls	Early non-invasive diagnosis	[58,59,60]
EryA-mCherry (CPE-specific probe)	Dental plaque	Specifically binds CPE without cross-reacting with host SM; discriminates healthy vs. periodontitis subjects	Non-invasive diagnostic tool	[61]
Specific SM species (d17:1/14:0, d16:1/15:0, d18:1/21:0)	Plasma	Positively correlated with periodontitis; negatively correlated with gingivitis	Differential diagnosis of periodontal diseases	[62]
Phosphorylated dihydroceramide	Diseased teeth, subgingival plaque	Detected at significantly elevated levels in periodontitis	Diagnostic biomarker	[63]
SphK1/S1P	Gingival crevicular fluid (GCF)	Elevated at periodontitis-affected sites; declines following non-surgical periodontal treatment	Treatment monitoring and therapeutic response evaluation	[71]
Serum S1P	Serum	Associated with periodontitis severity and extent; correlates with SphK1 expression in gingival epithelium and CD68^+^ macrophages	Systemic inflammatory marker linking periodontitis to chronic inflammation	[77]

This table summarizes sphingolipid-related biomarkers identified in periodontitis, including the biomolecule, sample type, key finding, and potential clinical application for each candidate marker. Abbreviations: CD68, cluster of differentiation 68; CPE, ceramide phosphoethanolamine; DH-CPE, dihydroceramide phosphoethanolamine; GCF, gingival crevicular fluid; S1P, sphingosine-1-phosphate; SM, sphingomyelin; SphK1, sphingosine kinase 1.

##### Bacterial Sphingolipids

Distinct from host-derived sphingolipids, periodontal pathogens produce structurally unique sphingolipids. Key periodontal pathogens such as Porphyromonas gingivalis and Tannerella forsythia synthesize CPE and its derivatives (DH-CPE and 2′-hydroxylated/acylated DH-CPE) [61]. These bacterial sphingolipids amplify inflammatory responses by activating the TLR2 signaling pathway and directly impairing bone regeneration processes, thereby contributing to persistent periodontal tissue destruction [61]. Unique non-mammalian dihydroceramides, including phosphoglycerol dihydroceramide (PGDHC) and phosphoethanolamine dihydroceramide (PEDHC), have also been identified in oral Gram-negative bacteria associated with chronic periodontitis, such as *P. gingivalis*, *T. forsythia* and *Prevotella intermedia* [59]. Among these molecules, PGDHC penetrates osteoclast precursor cells and increases lysosomal membrane permeability, leading to the release of cathepsin B (CatB) and enhanced enzymatic activity, which subsequently activates inflammatory and osteoclastogenic signaling pathways [78]. PGDHC also promotes the senescence-associated secretory phenotype through increased secretion of the pro-inflammatory cytokines TNF-α and IL-6, thus aggravating chronic periodontal inflammation [79]. Additionally, it downregulates key osteogenic genes such as Runx2 and ALP through the TLR2 pathway, indirectly promoting alveolar bone loss and contributing to periodontal tissue destruction [80,81]. These observations indicate that ceramide-related sphingolipids exert both pathogenic and regulatory effects in periodontitis, while ASMase maintains the balance of periodontal inflammation with bone metabolism.

PEDHC, a sphingolipid produced by Porphyromonas gingivalis, possesses structural features distinct from PGDHC. On the one hand, PEDHC reduces periodontal inflammatory infiltration and tissue damage by inhibiting the expression of NF-κB, MMP2, ADAM17, and IL-6, thus alleviating periodontal tissue destruction [40]. On the other hand, it induces intracellular ceramide accumulation by inhibiting acid ceramidase, which promotes epithelial cell apoptosis and inflammatory responses [40]. Therefore, these bacterial sphingolipid molecules participate in the pathological processes of periodontitis through pro-inflammatory and osteoclast-related mechanisms, providing new insights into tissue damage mediated by periodontal pathogens.

In summary, both host-derived and bacterial sphingolipids contribute to periodontitis through distinct mechanisms. Host ceramide and S1P primarily amplify inflammatory signaling and tissue destruction. In contrast, bacterial sphingolipids (CPE, PGDHC, and PEDHC) produced by *P. gingivalis* and *T. forsythia* exert pro-inflammatory and immunomodulatory effects that further shape host–pathogen interactions. The crosstalk between these two sphingolipid pools underscores the importance of metabolic homeostasis for periodontal health.

#### 3.2.3. Research on Preclinical Therapeutic Strategies for Periodontitis Targeting Sphingolipid Metabolic Pathways

From a therapeutic perspective, several studies have suggested that inhibiting ceramide synthesis or blocking ceramide-mediated pathological signaling pathways may effectively alleviate periodontal inflammation and bone loss [82]. Topical exogenous ceramide mitigates alveolar bone loss via CD300lf signaling in murine periodontitis models [66]. Its safety and efficacy within human periodontal tissues have yet to be verified, and favorable dermal safety cannot be extrapolated to intraoral application [66]. In addition, PEDHC influences host–pathogen interactions by modulating ceramide levels and inflammatory responses. These findings suggest that sphingolipid biosynthesis plays a key regulatory role in the virulence of *P. gingivalis* and that targeting key genes involved in bacterial sphingolipid synthesis may represent a potential therapeutic strategy. In addition, SphK1 inhibitors (e.g., DMS and SK1-I) and S1P receptor antagonists (e.g., VPC23019 and JTE013) attenuate inflammatory responses by suppressing S1P production and downstream signaling [70]. This strategy appears particularly relevant in periodontitis associated with diabetes or metabolic syndrome, suggesting that targeting SphK1 or S1PR may represent a promising therapeutic approach for this patient population [70]. Furthermore, Duarte et al. reported that the CatB inhibitor CA-074-ME markedly inhibits the osteoclastogenic effect of PGDHC, thereby providing a potential therapeutic target for preventing periodontitis-associated bone destruction [78]. Collectively, therapeutic strategies targeting key sphingolipid molecules, metabolic enzymes, receptors, and signaling pathways provide multiple potential directions for the development of precision therapeutic approaches for periodontitis. However, all evidence to date remains preclinical and requires further validation before clinical translation can be considered.

### 3.3. Sphingolipids and Oral Candidiasis

The oral cavity harbors a diverse microbiota that includes beneficial microorganisms, opportunistic commensals, and transient pathogens. *C. albicans* is a commensal fungus of the oral cavity that possesses pathogenic potential and can cause oral candidiasis under certain conditions. Increasing evidence suggests that sphingolipid metabolism is involved in *C. albicans*-associated oral diseases.

The main fungal sphingolipids include GlcCer and inositol phosphorylceramides (IPCs). GlcCer is a key component of plasma membrane lipid rafts crucial for maintaining membrane integrity and regulating signal transduction. Pharmacological inhibition of GlcCer significantly attenuates the virulence of *C. albicans* in a mouse model of systemic candidiasis without affecting its morphological transition or proliferative capacity [83]. Moreover, the acylhydrazone inhibitor D13 targets fungal GlcCer biosynthesis, exerts potent fungicidal activity against *C. albicans* and other pathogenic fungi, and alleviates systemic candidiasis in vivo [84]. IPCs are synthesized by inositol phosphorylceramide synthase (Ipc1). Ipc1 is a fungus-specific enzyme essential for fungal growth and lacks mammalian homologs [85]. Therefore, it represents a promising antifungal therapeutic target. In addition, phospholipomannan, a key glycosphingolipid of *C. albicans*, contains long β-1,2-mannose chains that interact with the host galectin-3/TLR2 complex [86,87,88]. This interaction activates the NF-κB signaling pathway while simultaneously impairing macrophage phagocytic function to facilitate chronic colonization and immune evasion of the fungus in the oral mucosa [89].

From a therapeutic perspective, aberrant sphingolipid metabolism is closely associated with antifungal resistance of *C. albicans*, making sphingolipid-targeted interventions a promising therapeutic strategy. Furthermore, loss of Ipc1 function or intracellular accumulation of long-chain sphingoid bases can alter fungal susceptibility to echinocandins [90]. In fluconazole-resistant clinical Candida isolates, the sphingolipid biosynthesis inhibitors myriocin and aureobasidin A exert synergistic antifungal activity when co-administered with fluconazole, robustly suppressing the growth of tested azole-resistant strains and offering a promising combinatorial therapeutic strategy to overcome fluconazole resistance [91].

Aureobasidin A (AbA) inhibits IPC synthase, suppressing sphingolipid synthesis and triggering ROS accumulation, mitochondrial damage, and apoptosis in *C. albicans* [92]. Topical AbA reduced tongue fungal load and inflammatory infiltration by 80% and 60%, respectively, after one week in murine oral candidiasis models, suggesting its potential as an oral therapeutic agent.

Cepharanthine–amphotericin B combination exerts synergistic inhibitory effects against *C. albicans*. It downregulates the plasma membrane gene PMP3 to block sphingolipid synthesis and impair membrane integrity [93]. This drug combination also suppresses hyphal development and biofilm formation, suggesting its potential use for oral mucosal infections.

Acylhydrazone compounds such as D13 and its derivative SB-AF-1002 target fungal GlcCer synthesis. In animal models of systemic candidiasis, D13 improved survival and demonstrated blood–brain barrier permeability with minimal mammalian toxicity [84]. SB-AF-1002, a more potent derivative, outperformed standard-of-care drugs in systemic models of candidiasis, cryptococcosis, and aspergillosis [94]. Furthermore, D13 showed synergistic activity with itraconazole, indicating that inhibition of GlcCer synthesis may restore azole sensitivity [84]. However, the efficacy of these compounds in oral mucosal infection models has not been evaluated. Additionally, the defensin RsAFP2 specifically binds to fungal GlcCer to inhibit pathogenic processes in *C. albicans* [95]. Moreover, the IgG2a monoclonal antibody, which targets fungal mannosylinositol phosphorylCER (MEST-3), exerts stronger inhibitory effects on IPC-related pathways compared to the anti-GlcCer antibody MEST-2 [96]. These findings suggest that inhibiting sphingolipid functions using specific monoclonal antibodies may represent a promising therapeutic strategy for fungal infections.

In summary, sphingolipid metabolism constitutes an important regulatory network influencing virulence and antifungal drug resistance in *C. albicans*. At the same time, key sphingolipid molecules and enzymes provide potential targets for therapeutic strategies against oral candidiasis, offering new mechanistic insights for improving antifungal treatment outcomes.

### 3.4. Sphingolipids and SS

SS, a chronic systemic autoimmune disease, is characterized by B cell-mediated autoantibody production and lymphocytic infiltration of exocrine glands [97]. However, the molecular mechanisms underlying the initiation and progression of SS remain incompletely understood. Sphingolipids provide valuable insights into the pathological mechanisms of SS and the development of targeted therapeutic strategies since they are essential structural components of cell membranes and important signaling molecules.

Sphingolipid components in the saliva and tears of patients with SS exhibit decreased ceramide and increased SM levels [98]. This alteration of the lipid barrier contributes to xerostomia and xerophthalmia, hallmark symptoms of SS [98]. Moreover, lipid alterations in saliva were consistent with findings from glandular biopsy samples, suggesting their potential value as biomarkers of glandular damage [98]. Furthermore, S1PR1 is highly expressed on inflammatory monocytes in patients with SS [99]. S1P signaling promotes CD4^+^ T-cell proliferation and IFN-γ secretion, thereby exacerbating lymphocytic infiltration of glandular tissues [99]. In addition, S1P enhances Fas expression and Fas-mediated caspase-3 activation in salivary gland epithelial cells, promoting apoptosis of acinar epithelial cells [99]. These processes disrupt the balance between pro-apoptotic sphingolipids (ceramide and sphingosine) and S1P-mediated pro-survival signaling, thus accelerating glandular damage. In NOD/LtJ mice, a spontaneous primary SS model, elevated BMP6 expression in salivary glands upregulates Cers6, driving ceramide accumulation and NF-κB-dependent Th1 polarization [100]. This BMP6–ceramide–Th1 axis represents a direct mechanistic link between sphingolipid dysregulation and the autoimmune pathology of SS.

In NOD/LtJ mice, pharmacological inhibition of ceramide synthesis with myriocin reduced Th1 and Th17 cell infiltration in salivary glands, restored expression of the water channel proteins Aqp5 and Nkcc1, and partially rescued salivary flow [100]. These findings provide direct in vivo evidence that inhibiting ceramide synthesis may attenuate SS-associated glandular dysfunction.

In NOD mice, a spontaneous animal model of SS, sphingosylphosphorylcholine elevates the abundance of regulatory B cells within salivary gland tissues and splenocytes. This treatment preserves salivary secretion and alleviates inflammatory cell infiltration [101]. In vitro experiments using HSG human salivary gland epithelial cells corroborated in vivo findings: sphingosylphosphorylcholine upregulated salivary secretion-associated genes such as M3R and AQP5 under inflammatory stimulation [101]. The Chinese herbal formulation FuFang Runzaoling improves inflammatory responses in SS by modulating the gut microbiota–sphingolipid axis, suggesting a potential integrative therapeutic strategy combining traditional Chinese and Western medicine approaches [102].

In summary, disrupted sphingolipid metabolism serves as a key inducer of immune activation and glandular injury in SS. Given this, sphingolipid metabolic cascades and the S1P/S1PR1 signaling axis hold great potential as therapeutic targets. Future research needs to further elucidate the functions of distinct sphingolipid species and their intricate regulatory networks to facilitate the development of more accurate diagnostic tools and refined targeted therapies for SS.

### 3.5. Sphingolipids and Periapical Diseases

Emerging preliminary data indicate that bacterial sphingolipids are likely implicated in periapical diseases pathogenesis. Phosphorylated dihydroceramides derived from *P. gingivalis* and Porphyromonas endodontalis have been detected in infected root canals [103]; in vitro assays demonstrate these lipids trigger PGE_2_, IL-6, and TNF-α secretion through TLR2-mediated signaling [104,105]. Moreover, bacterial sphingolipids suppress osteoblast differentiation and boost osteoclastogenesis [106]. Notably, most mechanistic observations above originate from periodontitis models, and their direct relevance to periapical bone should be validated experimentally using dedicated periapical diseases models.

Two recent metagenomic analyses have revealed functional signatures of sphingolipid-associated pathways in periapical lesions. Georgiou et al. identified enrichment of sphingolipid signaling pathways in the root canal microbiome of patients with asymptomatic apical periodontitis [107]. A subsequent study by Pérez-Carrasco et al. identified significant enrichment of sphingolipid signaling and catabolic pathways in large periapical lesions (>7 mm) versus small lesions (<3 mm). The authors hypothesized that bacterial sphingomyelinase hydrolyses host sphingomyelin to produce ceramide and its downstream mediators (C1P and S1P), amplifying inflammatory cascades and driving lymphocyte recruitment to periapical tissues [108]. These observations imply a correlation between microbial sphingolipid pathway activity and lesion progression. Importantly, both datasets rely on predictive metagenomic profiling; direct quantification of sphingolipid concentrations within diseased periapical tissue is lacking.

## 4. Limitations and Future Perspectives

Despite significant advances in understanding sphingolipid metabolism in oral diseases, several important research gaps remain. The biological functions of atypical sphingolipids, such as 1-deoxysphingolipids, and the regulatory roles of sphingolipid catabolic pathways are still incompletely understood, and the functions of several key molecules require further experimental validation. For example, the therapeutic potential of SphK2 in cancer remains unclear, and its applicability as a therapeutic target for OSCC has not yet been systematically investigated [109]. In addition, most currently proposed sphingolipid biomarkers remain at the basic research stage, lacking validation in large clinical cohorts. In contrast, the standardization and clinical feasibility of detection methods still require further optimization. Similarly, many therapeutic strategies targeting sphingolipid metabolism remain limited to in vitro experiments and animal models. For instance, FTY720′s therapeutic efficacy has been evaluated in only a single OSCC study [47], while the clinical applicability of mitochondria-targeted ceramide derivatives, characterized primarily in non-OSCC HNSCC models, remains to be validated in oral cavity-specific tumor models. Moreover, although nanodelivery systems show promise for improving the targeting efficiency and bioavailability of sphingolipid-based drugs, their development remains at an early preclinical stage, and several technical problems, including scalability, batch-to-batch reproducibility, and tumor-specific targeting in vivo, must be addressed before clinical evaluations in OSCC can be considered [50,109].

Future research on sphingolipid metabolism in oral diseases may focus on several key directions. First, integrated multi-omics approaches, including lipidomics, transcriptomics, and proteomics, could be used to systematically characterize sphingolipid metabolic profiles in different oral diseases and clarify the regulatory networks and biological functions of key molecules. Second, efforts should focus on translational studies of sphingolipid biomarkers, including the development of standardized detection systems and combined biomarker panels to improve diagnostic accuracy and prognostic assessment in oral diseases. Third, further work is needed to develop precise therapeutic strategies targeting sphingolipid metabolism, including optimizing existing inhibitors and receptor antagonists, exploring combination therapies (e.g., integrating sphingolipid inhibitors with radiotherapy, chemotherapy, or immunotherapy), and improving drug delivery through nanotechnology-based approaches. In addition, investigating the relationship between sphingolipid metabolism, oral microbiota, and systemic diseases, such as diabetes mellitus and autoimmune diseases, may provide new perspectives for interdisciplinary diagnosis and treatment of oral diseases.

This review has several limitations. First, the lipidomic studies covered herein have adopted disparate analytical detection platforms and inconsistent data reporting criteria, which hinders direct comparison of results between different studies. Second, most clinical trials had limited sample sizes. Moreover, numerous mechanistic insights are drawn from preclinical animal models or non-oral cell lines, which fail to fully mimic the pathological characteristics of oral disorders in humans. All the above limitations should be considered when evaluating the clinical translational value of the evidence summarized in this review.

## 5. Conclusions

**Figure 2:**
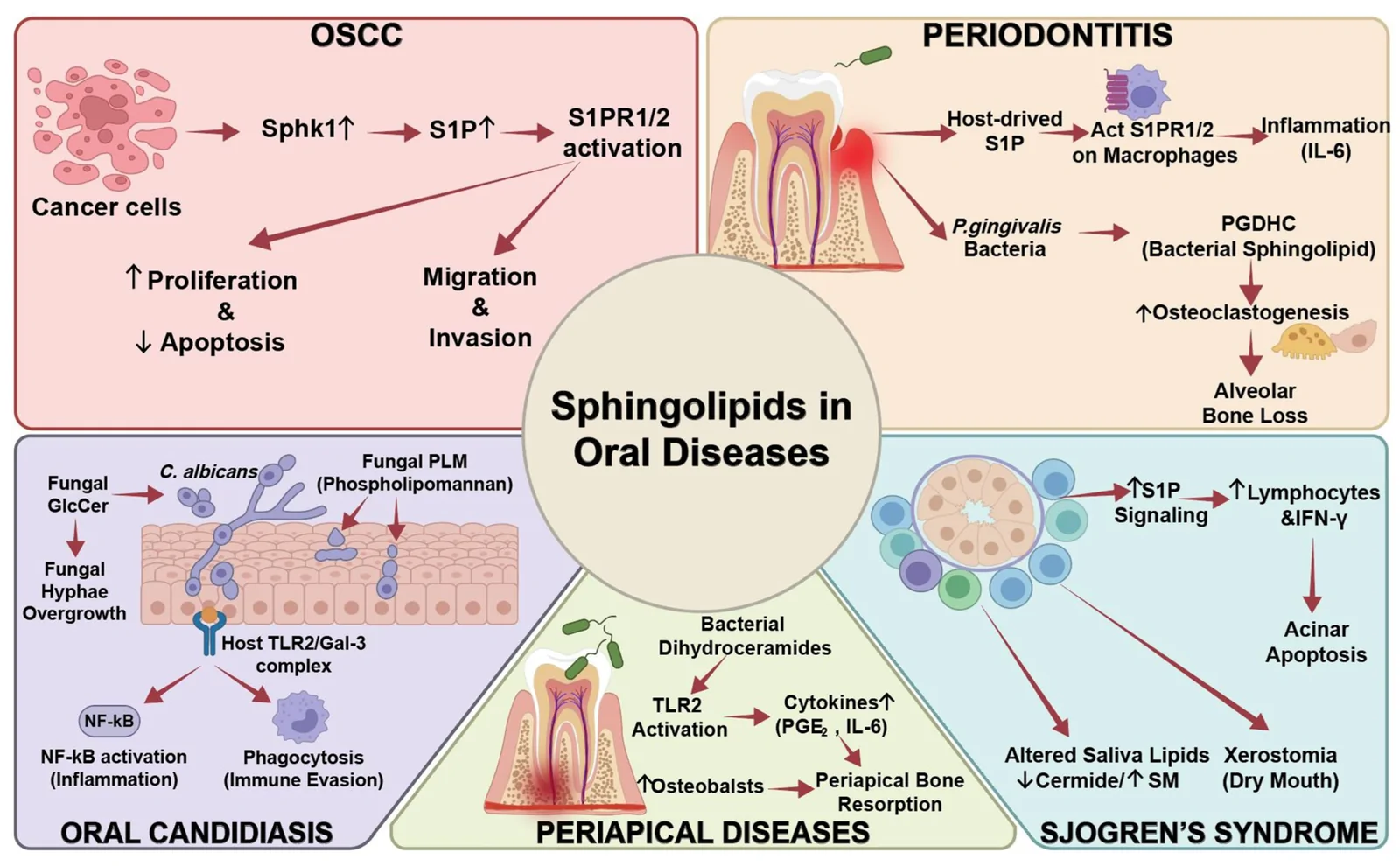
Contributions of Sphingolipids and Their Metabolites to Oral Disease Pathophysiology. Integrated schematic illustrating the central pathogenic roles of dysregulated sphingolipid signaling across five major oral disorders: OSCC, periodontitis, oral candidiasis, periapical diseases and SS. In OSCC, elevated SphK1-S1P-S1PR1/2 signaling drives tumor proliferation, migration and inhibits apoptosis. In periodontitis, host-derived S1P activates macrophage inflammatory cascades, while Porphyromonas gingivalis-derived bacterial PGDHC triggers osteoclastogenesis and alveolar bone loss. In oral candidiasis, fungal GlcCer and phospholipomannan (PLM) interact with host TLR2/Galectin-3 to induce NF-κB-mediated inflammation and immune evasion. Bacterial dihydroceramides activate TLR2 signaling to promote periapical inflammatory bone resorption. In Sjögren’s syndrome, altered salivary ceramide/sphingomyelin balance and excessive S1P signaling trigger glandular inflammation and acinar epithelial apoptosis.

Sphingolipids are essential structural components of cell membranes and important signaling molecules that contribute to maintaining oral physiological homeostasis. Dysregulation of sphingolipid metabolism, together with the abnormal activation of related molecules, enzymes, and receptors, is involved in the pathogenesis of multiple oral diseases, including OSCC, periodontitis, SS, oral candidiasis, and periapical diseases (Figure 2). Clarifying the complex regulatory network of sphingolipid metabolism will provide novel candidate biomarkers and promising therapeutic targets for the prevention, early diagnosis, and precise treatment of these oral disorders.

## Figures and Tables

**Table 1:** Sphingolipid-targeted Therapeutic Strategies for OSCC: Preclinical Evidence.

Target	Therapeutic Strategy/Drug	Key Effect	Experimental Model	Evidence Level	Refs.
SphK1	Pharmacological inhibition; gene silencing (siRNA)	Suppresses OSCC progression; partially restores radiosensitivity	Radioresistant OSCC cell lines (SCC-15, SCC-25)	In vitro	[44,45]
SphK2	QZQ-01115 (novel SphK2 inhibitor)	Suppresses CAL-27 proliferation (IC50 = 5.84 μM); induces G0/G1 arrest and apoptosis via Bcl-2/Bax; disrupts S1P/ceramide balance	CAL-27 cell line; xenograft mouse model	In vitro + In vivo	[46]
Acid ceramidase	N-Oleoylethanolamine (NOE); gene silencing	May reverse cisplatin resistance in HNSCC	HNSCC cell lines	In vitro	[23]
S1PR2	JTE013 (S1PR2 antagonist)	May enhance EGFR-targeted therapy and suppress OSCC metastasis	OSCC cell lines	In vitro	[30]
S1PR3	CAY10444 (S1PR3 antagonist); genetic knockdown	Impairs AKT/WEE1 cell cycle checkpoint; suppresses OSCC proliferation	OSCC cell lines; in vivo tumor models	In vitro + In vivo	[42]
S1PR modulator	FTY720 + paclitaxel	Reduces viability, clonogenicity, and CSC sphere formation in cisplatin-resistant OSCC cells; inhibits xenograft tumor growth via NF-κB/SET/SphK1 suppression	Cisplatin-resistant OSCC cells (Cal27, SCC9); xenograft mouse model	In vitro + In vivo	[47]
Ceramide	C6-ceramide + PKC412	Synergistically enhances anti-tumor effects via Akt-mTOR pathway inhibition and Mcl-1 downregulation	HNSCC preclinical models	In vitro	[48]
Ceramide (liposomal)	Liposomal C6-ceramide formulations	Engineered to address ceramide delivery constraints; anti-osteosarcoma activity demonstrated	Osteosarcoma cell lines; xenograft model (non-OSCC)	In vitro + In vivo	[49]
SphK1/Ceramide (nanodelivery)	Gold nanorod-mediated SphK1-siRNA; ceramide nanoliposomes	Explored for improving drug delivery and tumor targeting; development at early preclinical stage	HNSCC cell lines	In vitro (early preclinical)	[50]

This table summarizes sphingolipid-targeted therapeutic strategies investigated in OSCC, including the molecular target, therapeutic agent, key antitumor effects, experimental model system, and level of evidence. Abbreviations: CSC, cancer stem cell; EGFR, epidermal growth factor receptor; HNSCC, head and neck squamous-cell carcinoma; OSCC, oral squamous-cell carcinoma; S1P, sphingosine-1-phosphate; SphK, sphingosine kinase; S1PR, S1P receptor.

## Data Availability

No new data were created or analyzed in this study.

## Abbreviations

The following abbreviations are used in this manuscript:

AbA	Aureobasidin A
ASMase	Acid sphingomyelinase
C1P	Ceramide-1-phosphate
CatB	Cathepsin B
CER	Ceramide
CerS1–6	Ceramide synthases 1–6
CERT	Ceramide transfer protein
CPE	Ceramide phosphoethanolamine
CSC	Cancer stem cell
DEGS1	Dihydroceramide desaturase 1
DES	Dihydroceramide desaturase
DH-CPE	Dihydroceramide phosphoethanolamine
DHSph	Dihydrosphingosine
EGF	Epidermal growth factor
EGFR	Epidermal growth factor receptor
EMT	Epithelial–mesenchymal transition
ER	Endoplasmic reticulum
GCF	Gingival crevicular fluid
GCS	Glucosylceramide synthase
GlcCer	Glucosylceramide
HNSCC	Head and neck squamous-cell carcinoma
Ipc1	Inositol phosphorylceramide synthase
IPC	Inositol phosphorylceramide
KDS	3-Ketodihydrosphingosine
KDSR	3-Ketodihydrosphingosine reductase
LPP1–3	Lipid phosphate phosphatases 1–3
LPS	Lipopolysaccharide
OSCC	Oral squamous-cell carcinoma
PEDHC	Phosphoethanolamine dihydroceramide
PGDHC	Phosphoglycerol dihydroceramide
ROS	Reactive oxygen species
S1P	Sphingosine-1-phosphate
S1PR1–5	Sphingosine-1-phosphate receptors 1–5
SM	Sphingomyelin
SMase	Sphingomyelinase
SphK1	Sphingosine kinase 1
SphK2	Sphingosine kinase 2
SPL	S1P lyase
SPP1	S1P-specific phosphatase 1
SPP2	S1P-specific phosphatase 2
SPT	Serine palmitoyltransferase
SS	Sjögren’s syndrome
TLR2	Toll-like receptor 2
